# Recurrent Peripheral Artery Disease in a 45-Year-Old Male Without Hypercoagulable Comorbidities

**DOI:** 10.7759/cureus.89057

**Published:** 2025-07-30

**Authors:** Adi Cohen, Jessica Cohen, Igor Cordeiro de Oliveira, Sheela Anasseri, Faryal Ahmed, Rebecca Cherner

**Affiliations:** 1 Medicine, Nova Southeastern University Dr. Kiran C. Patel College of Osteopathic Medicine, Fort Lauderdale, USA; 2 Family Medicine, Broward Health Medical Center, Fort Lauderdale, USA

**Keywords:** atherosclerosis, comorbidities, hypercoagulability, peripheral artery disease, substance abuse

## Abstract

Peripheral artery disease (PAD) is a progressive vascular disease characterized by atherosclerotic narrowing of peripheral arteries, resulting in decreased blood flow to the extremities. Common risk factors for PAD are diabetes, hypertension, and hyperlipidemia, although it can also occur in patients without these comorbidities, such as in the use of marijuana or alcohol. Recent research suggests that marijuana and alcohol use lead to endothelial dysfunction and vascular inflammation, conditions found in PAD. We present the case of a 45-year-old male who presented with complaints of left lower leg pain for three months. His past medical history includes PAD involving a left femoral to popliteal arterial bypass surgery in 2017 and a saphenous vein graft revision in 2018. The patient’s social history was positive for polysubstance abuse with daily marijuana and alcohol use for the past 20 years. While in the emergency department (ED), a left lower extremity arterial Doppler duplex ultrasound procedure was performed and demonstrated severe atherosclerotic disease with occlusion of the mid and distal superficial femoral artery, popliteal artery, and dorsalis pedis artery. Three days after admission to the ED and following cardiology clearance, the patient underwent a left femoral-tibial arterial bypass with autogenous reverse saphenous graft due to a left femoral and popliteal artery occlusion. This case addresses a potential association between chronic substance use and PAD with a lack of classical risk factors. Although the vascular effects of alcohol and marijuana have been acknowledged in the literature, they remain unrecognized by many physicians. This case highlights the need for increased awareness of potential non-traditional risk factors for PAD, in recognition of earlier intervention and improved patient outcomes.

## Introduction

Peripheral artery disease (PAD) is a vascular condition characterized by decreased blood flow resulting from narrowing of the arteries that supply the extremities [1]. The most common cause is atherosclerosis, a buildup of plaque composed of fat and cholesterol that deposits in the walls of peripheral arteries [1,2]. Other less prevalent causes of PAD include irritation of these arteries, injury to the extremities, and radiation exposure [1]. The arterial narrowing significantly reduces the amount of blood available to supply the tissues and muscles of a limb. A 50% decrease in arterial diameter corresponds to a 75% loss of cross-sectional area, which receives limited blood flow [3]. As a form of compensation, blood flow shifts towards smaller arterial branches; however, these vessels carry less than what is needed for limb viability [3].

The clinical presentation of PAD can be asymptomatic; however, the most common symptom includes limb claudication characterized by pain, muscle cramping, numbness, or weakness in the extremities during physical activity [1]. If left untreated, the lack of blood flow over an extended period can lead to tissue death, commonly referred to as gangrene. This irreversible consequence of PAD can require amputation if blood flow isn’t restored [2]. On physical exam, the affected limb may present as cool to the touch with diminished or absent peripheral pulses [1]. There may also be associated pallor, muscle atrophy, hair loss, pain on palpation, ulcers or sores that will not heal, and the presence of a bruit [1-3]. For the diagnosis of PAD, the ankle-brachial index (ABI) is utilized as a measure of lower extremity arterial perfusion [3]. The ABI is calculated by dividing the systolic blood pressure at the ankle by the systolic blood pressure at the arm. A value less than 0.9 is diagnostic of PAD [2,3].

Risk factors for PAD include age > 65, obesity, smoking, family history, diabetes, hypertension, hypercholesterolemia, hyperhomocysteinemia, sedentary lifestyle, and a diet rich in cholesterol and saturated fat [1-3]. Of these, smoking has been found to have the most significant effect on disease severity, leading to a four times greater risk of developing PAD with a higher likelihood of progression to limb ischemia and amputation [3]. The following case report discusses a patient with a history of marijuana use for 20 years who lacked other hypercoagulable comorbidities and presented with lower leg pain secondary to PAD.

## Case presentation

We present a 45-year-old male who arrived at the emergency department (ED) with complaints of left lower leg pain that has symptomatically occurred over the past three months. Past medical history includes PAD status post left femoral to popliteal arterial bypass surgery in 2017, which was then revised with a saphenous vein graft in 2018. The patient also suffers from polysubstance abuse, with daily marijuana and alcohol use for the past 20 years. The pain was described as a stiffness that began at the knee and radiated down the entire left leg to the toes, with a predominant localization in the left calf and shin. During this admission, the patient rated the pain an 8/10 on the pain scale and stated that it had been significantly worsening over the past few months. The pain was alleviated by lying down and aggravated by ambulation and movement. He compared the pain to the same sensation felt before his peripheral artery bypass years ago. Since the patient’s previous peripheral artery bypass, he has been under the care of vascular surgery, who prescribed atorvastatin, aspirin, clopidogrel, and apixaban to be taken daily. The patient claims he has been medication non-compliant since October 2024, secondary to a lack of insurance and an inability to follow up with the physician to obtain a refill.

Upon primary evaluation, his vital signs were stable: afebrile at 36.6°C, normotensive at 118/72 mmHg, and SpO2 at 94% on room air. He exhibited moderate tenderness to palpation along the left calf; however, his dorsalis pedis and posterior tibial pulses were palpable. The motor, sensory, and skin exams were insignificant. Initial laboratory findings showed lipid and coagulation studies within normal range. An autoimmunity panel, as well as inflammatory markers, was unremarkable. Additional pertinent laboratory values are included in Table 1.

**Table 1:** Patient's lab values on initial assessment RBC: red blood cells, MCV: mean corpuscular volume, PTT: partial thromboplastin time, PT/INR: prothrombin time/international normalized ratio, LDL: low-density lipoprotein, HDL: high-density lipoprotein, HbA1c: glycated hemoglobin, vWF Ag: von Willebrand factor, AST: aspartate aminotransferase, ALT: alanine aminotransferase

Lab test	Reference range	Initial patient values
RBC (10⁶/uL)	4.30-5.80	4.89
Hemoglobin (g/dL)	13.0-17.3	15.7
Hematocrit (%)	38.0-52.0	45.3
MCV (fL)	78.0-100.0	92.6
Platelets (10³/uL)	140-400	164
Random glucose (mg/dL)	70-105	119
PTT (sec)	24.6-35.3	29.4
Protime (sec)	12-14.4	13.3
PT/INR (sec)	≤4.9	1.0
Triglycerides (mg/dL)	0-130	52
Cholesterol (mg/dL)	≤200	174
LDL (mg/dL)	≤100	79
HDL (mg/dL)	40-60	85
HbA1c (%)	4.0-6.0	5.3
Fibrinogen (mg/dL)	208-484	254
vWF Ag (%)	50-217	167
Factor VIII clotting activity (%)	50-180	149
Total bilirubin (mg/dL)	0.2-1.2	0.4
Alkaline phosphatase (U/L)	40-150	78
AST (U/L)	5-34	49
ALT (U/L)	≤55	17

**Figure 1:**
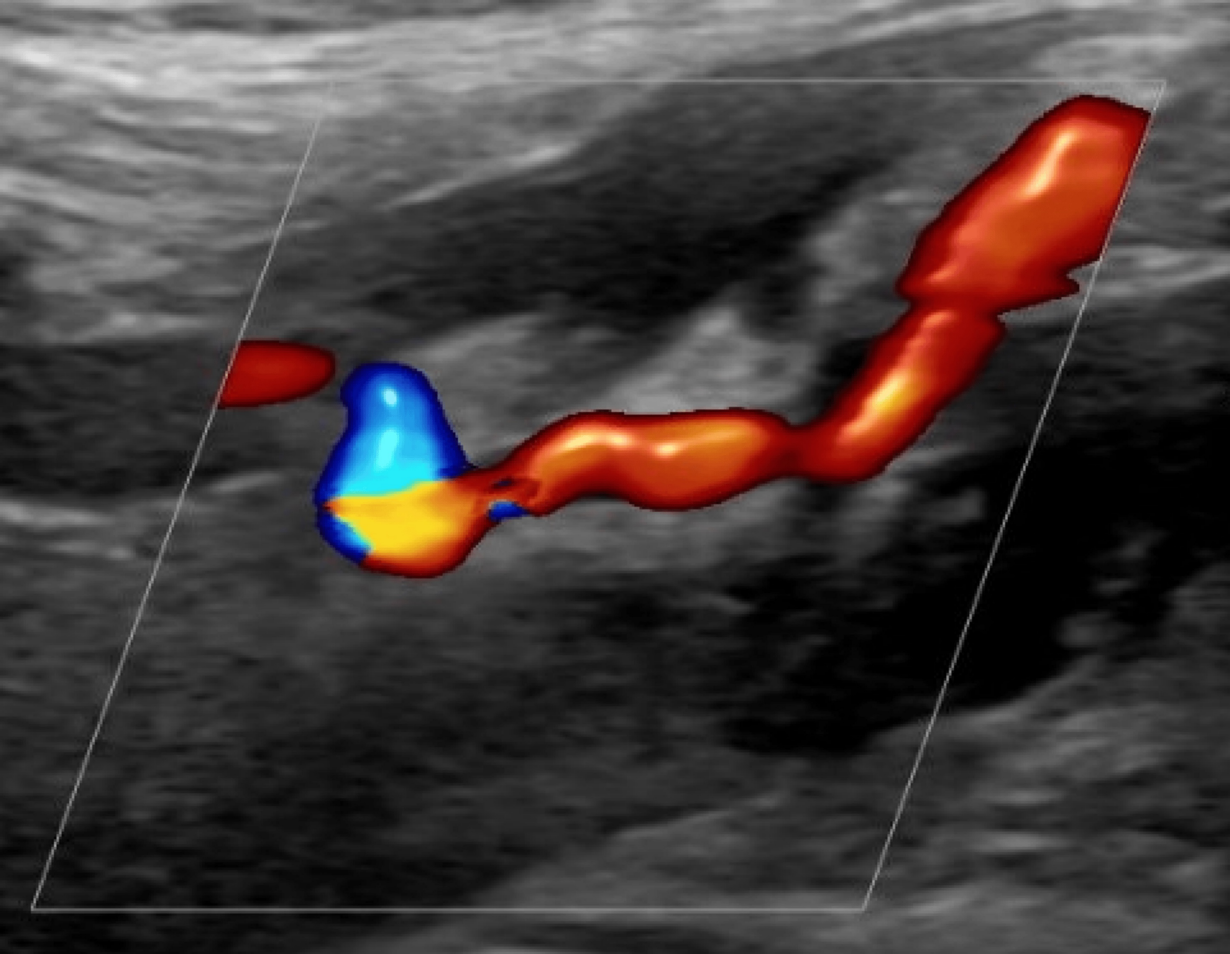
Left lower extremity arterial Doppler duplex ultrasound demonstrating severe atherosclerotic disease with occlusion of the mid and distal superficial femoral artery, popliteal artery, and dorsalis pedis artery

While in the ED, a left lower extremity arterial Doppler duplex ultrasound was performed and demonstrated severe atherosclerotic disease with occlusion of the mid and distal superficial femoral artery, popliteal artery, and dorsalis pedis artery (Figure 1). A left lower extremity venous duplex ultrasound showed no sonographic evidence for deep venous thrombosis. The patient received lorazepam, clonidine, ondansetron, aspirin, clopidogrel, and morphine. Family medicine admitted the patient to their service, and vascular surgery was consulted.

**Figure 2:**
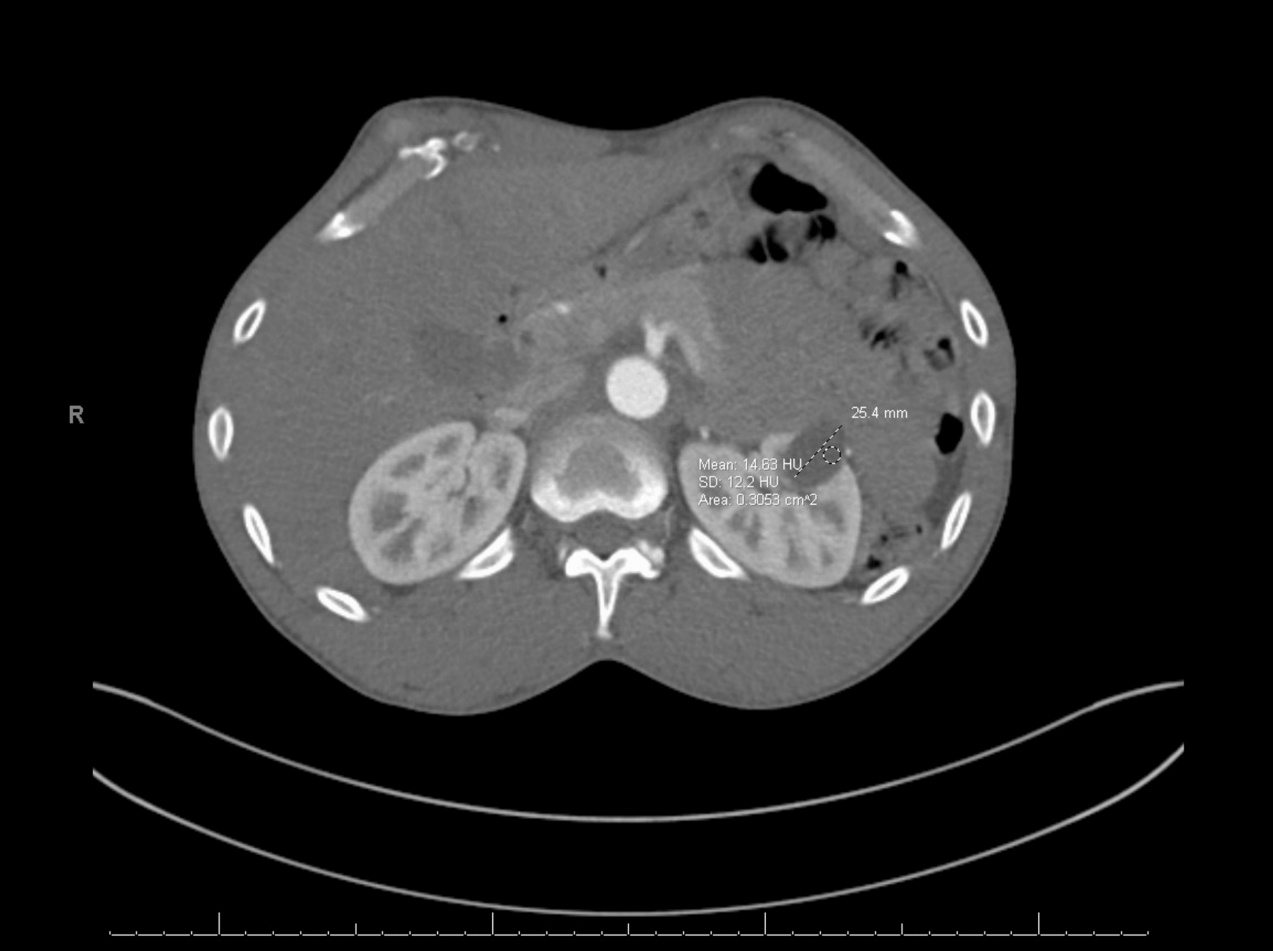
CT angiogram of the aorta and bilateral iliofemoral arterial runoff, which showed an indeterminate low-density lesion in the left kidney measuring 2.5 cm, indicative of a benign left renal cyst CT: computed tomography

Once admitted to inpatient care, additional imaging was ordered to obtain a thorough clinical picture. Bilateral carotid artery duplex ultrasound showed no hemodynamically significant narrowing of the internal and common carotid arteries. Vascular surgery requested a CT angiogram of the aorta and bilateral iliofemoral arterial runoff, which revealed an indeterminate, low-density lesion in the left kidney measuring 2.5 cm, consistent with a benign left renal cyst (Figure 2). Right lower extremity vein mapping ultrasound also demonstrated a patent greater saphenous vein as a potential graft.

Three days after admission to the ED and following cardiology clearance, the patient underwent a left femoral-tibial bypass with autogenous reverse saphenous vein graft due to a left femoral and popliteal arterial occlusion. Postoperatively, the patient complained of bilateral lower extremity pain with vitals presenting as hypertensive at 150/90 mmHg and afebrile at 36.9°C with 95% O₂ saturation on room air. Neuromuscular checks were performed every two hours by vascular surgery with adequate pain management.

Two days postoperative, the patient spiked a fever at 100.6°F, which resolved with antipyretics and was thought to be due to a systemic inflammatory response syndrome reaction following the procedure. He also began saturating in the 80-90% range, so a nasal cannula was initiated, which was discontinued 24 hours later. The patient steadily improved, worked with physical therapy on ambulation, and received patient education on wound care. Additionally, the drains placed during the surgical procedure were removed, and pain management was optimized. Discharge occurred eight days after admission and five days postoperatively, with the medications of aspirin and Plavix, patient education, outpatient referral to physical therapy, wound care supplies, a rolling walker, return to ED precautions, and follow-up instructions. Outpatient follow-up with vascular surgery as well as primary care was made for the patient one week postoperatively.

## Discussion

Lower extremity PAD is a concern in patients with significant risk factors, including age >65, hyperlipidemia, hypertension, diabetes mellitus, chronic kidney disease, and smoking [4]. Those with three or more of these risk factors have a 10-fold increased risk of developing PAD [4]. However, our patient presented with a 20-year history of cannabis and alcohol use and no other significant risk factors for PAD.

The term cannabis arteritis (CA) was first mentioned in the 1960s and is thought to be a less recognized potential cause of PAD in younger patients [5]. Since then, over 50 case studies have been published, including one of a 38-year-old male with a past medical history only significant for chronic cannabis use who developed a dry necrotic lesion of the left big toe [5,6]. Further imaging studies and evaluation of this 38-year-old revealed arterial disease in the lower extremities, predominantly on the left side, in the absence of any significant atherogenic or thrombogenic risk factors, which is similar to the patient presented in our case report [6].

Marijuana is thought to impact the cardiovascular system through its biologically active ingredients, which include cannabinoids and 9-tetrahydrocannabinol (THC) [5]. THC binds to cannabinoid receptors in the body. Cannabinoid 1 (CB1) receptors are expressed in multiple tissues, including the vascular endothelium, while cannabinoid 2 (CB2) receptors are expressed by immune cells and the cardiovascular system [7]. The mechanism of CA is still not completely understood; however, stimulation of the CB1 and CB2 receptors has been shown to have an impact on vessel walls that could contribute to the formation of atherosclerotic disease [7,8]. Specifically, it has been demonstrated that CB1 receptor agonists induce a proatherosclerotic cascade through their effects on endothelial cells, lipid metabolism, and vascular smooth muscle cells, whereas CB2 receptor agonists promote an anti-atherogenic cascade [8].

Cannabis use remains under investigation for significant ties to increased risk of future cardiovascular events, especially in younger patients. One study analyzed de-identified patient information from more than 30 million patients in the National Inpatient Sample from 2016 to 2019. This study found that individuals who used cannabis were three times more likely to develop PAD, but without any significant increased risk in mortality [9]. A cross-sectional study that utilized 2016 to 2020 data from the Behavioral Risk Factor Surveillance Survey from 27 American states and two territories showed that cannabis use in young adults (men aged <55) significantly correlated with an increased risk of coronary heart disease, myocardial infarction, and stroke [10]. Finally, another retrospective study focusing on adults less than or equal to 50 years of age, utilizing 2010 to 2018 data from the TriNetX Health Research Network, provided more evidence on marijuana use as an independent risk factor for adverse cardiovascular events, even in populations without other traditional cardiovascular risk factors [11].

In addition to the debate of potential effects of chronic marijuana use on the cardiovascular system, our patient also raised the discussion of restenosis occurrence in the same extremity seven years after a previous bypass surgery. A study following patients undergoing lower extremity bypass (LEB) at one of the academic centers in the Vascular Study Group of New England between 2003 and 2011 found that the number of patients undergoing a second LEB increased from 22% to 38% [12]. The same study also found that patients undergoing a second bypass operation were more likely to be younger and to have a higher rate of smoking [12]. Further, findings showed that patients undergoing a second LEB were more likely to be prescribed a statin, clopidogrel, or aspirin in the preoperative period [12]. Our case report discusses a young male with continued chronic marijuana use following his first bypass surgery on a medication regimen of Plavix, atorvastatin, and aspirin, which are all factors associated with higher chances of a second LEB.

Unfortunately, he also faced challenges with medication coverage by insurance, hindering him from adhering to his medications after his first bypass surgery. Given this, further research is necessary to evaluate cannabis use and its impact on atherosclerotic disease and restenosis, as well as the effects of medical non-compliance on the outcomes of a second surgical intervention. More information on these factors can help improve patient education, preventative care, and surgical management in patients with PAD.

## Conclusions

This case evaluates the role of chronic marijuana and alcohol use and their association with PAD in the absence of other risk factors. While PAD is associated with diabetes, hypertension, and hyperlipidemia, new research suggests that chronic substance use may contribute to vascular dysfunction and atherosclerotic disease. Doctors must remain vigilant of the diagnosis of PAD, particularly in patients presenting with signs and symptoms such as exertional limb pain or ischemia. Further research and a multidisciplinary approach are necessary to develop effective strategies for recognizing and managing PAD, thereby preventing delays in care and improving patient outcomes.
